# Vagal Nerve Stimulation Device-Induced Second-Degree Heart Block and Sinus Pauses

**DOI:** 10.7759/cureus.87722

**Published:** 2025-07-11

**Authors:** Megan DeJong, Badria Munir, Farman Ali, Eric J Seachrist

**Affiliations:** 1 Neurology, West Virginia University School of Medicine, Morgantown, USA; 2 Neurology, Mayo Clinic Alix School of Medicine, Jacksonville, USA

**Keywords:** arrhythmia, epilepsy, second degree heart block, vagal nerve stimulator, vns

## Abstract

A 41-year-old male with a history of preterm birth, intellectual disability, and refractory epilepsy on multiple anti-seizure medications with an active vagus nerve stimulation device (VNS) presented for progressive lethargy and a global decline in health. The symptoms began two years after placement of a second VNS, as the first was removed years earlier due to infection complications. At the time of the lethargy and global decline presentation, the VNS was set at an output of 3.25 mA. On admission to the hospital, telemetry showed frequent sinus pauses lasting 2 to 8.5 seconds. An electrocardiogram (EKG) showed a second-degree Mobitz type II atrioventricular block. A transthoracic echocardiogram showed an ejection fraction of 65% without valvular abnormalities. A sleep study showed mild sleep apnea. The electroencephalogram (EEG) did not show epileptiform activity correlated to these events. Previous workup included a transthoracic echocardiogram and a 30-day cardiac monitor, which had shown normal sinus rhythm. Complete resolution of sinus pauses occurred after the VNS device was turned off. Cardiac arrhythmias associated with VNS are exceedingly rare and can occur years after implantation. High clinical suspicion is required in patients with VNS devices who develop new-onset lethargy and/or atonic episodes without other causes. This case aligns with previously reported cases and provides more evidence of these rare but potentially life-threatening events.

## Introduction

This article was previously presented as an abstract at the 2023 American Academy of Neurology Annual Scientific Meeting on April 23, 2024 [1]. Vagal nerve stimulation (VNS) devices are an adjunctive therapy for medically refractory epilepsy in adults and children, and over 125,000 devices have been implanted worldwide [2,3]. Indications for VNS consideration typically include medically refractory focal or generalized epilepsy in which surgical resection is not an option or previous resection was unsuccessful [4]. VNS can reduce seizure frequency by 50% and become more effective with longer-term use [4].

A VNS is a battery-operated device placed near the carotid bifurcation, below the left collarbone, with its coils wrapped around the left vagus nerve. The battery life is estimated at six to eleven years, depending upon user settings and the model utilized [5]. The device delivers electrical impulses to the vagus nerve via stimulating leads. Left-sided VNS placement is recommended, as the right vagus nerve innervates the sinoatrial node and is directly involved in maintaining the cardiac rhythm. Moreover, right VNS placement has resulted in respiratory depression in children and is thus not recommended, unless the left-sided approach is not an option [6]. However, not an absolute contraindication, patients with underlying cardiac disorders are at risk of developing arrhythmias and are therefore not typically candidates for vagus nerve stimulation [4].

The mechanism of action of VNS remains unclear: it is thought to use intermittent stimulation of the vagus nerve to suppress seizure frequency. The most common side effects seen with vagal nerve stimulation include cough, hoarseness, and shortness of breath [3]. A long-term follow-up study of 454 VNS clinical trial patients demonstrated device tolerability; furthermore, a sample of these patients underwent Holter monitoring two years after VNS placement, and only two asymptomatic dysrhythmias were reported: bradycardia and supraventricular tachycardia [3]. Here, we report a case of second-degree heart block that occurred four years after placement of a VNS device, with presenting symptoms of syncopal events and rapidly progressive lethargy.

## Case presentation

We have a 41-year-old patient with a past medical history significant for preterm birth, intellectual disability, major depressive disorder, and refractory focal epilepsy. At the time of presentation, he was on multiple anti-seizure medications (ASMs), including zonisamide, eslicarbazepine, brivaracetam, clonazepam, and recently added cenobamate. His seizure semiology entails multiple types, including both epileptic and non-epileptic episodes. Epileptic spells were characterized by brief nocturnal, as well as awake, episodes of hypermotor activity, nonsensical speech, periods of generalized shaking, and bladder and bowel incontinence without associated stiffening of the whole body. Lastly, the patient experienced occasional non-epileptic episodes of staring spells without automatisms and repetitive motor behaviors. These spells were captured on video EEG and cardiac telemetry without any electrographic correlation. He underwent left-sided VNS placement at the age of 24, but the VNS device had to be removed at age 32 due to a bacterial infection at the VNS site. The VNS was placed again on the left side at the age of 38 due to increasing frequency of seizures and poor control with medications. Soon after placement of the VNS, the patient began to have new spells during awake states of sudden loss of whole-body tone with subsequent collapse.

Assuming a new seizure type, the patient was admitted to the epilepsy-monitoring unit, and events were captured on continuous EEG but did not correlate with epileptiform discharges. Subsequently, an extensive cardiac workup was pursued, including EKG, 30-day cardiac monitoring, and a transthoracic echocardiogram, which did not demonstrate cardiovascular causes. Those spells were resolved spontaneously. The etiology for these events remained unknown, although a transient side effect of VNS was presumed; furthermore, since the episodes resolved, no VNS adjustment or removal was required. Two years later, after initiation of cenobamate, the patient started to experience worsening lethargy and a global decline in his general condition. His cenobamate dose was reduced to assess for lethargy secondary to the new medication; however, continued excessive drowsiness prompted another hospital admission.

**Figure 1:**
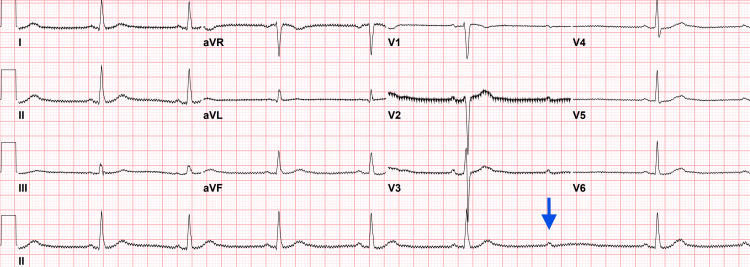
EKG showing sinus bradycardia with second-degree AV block Sinus bradycardia with a suspected second-degree, Mobitz type II atrioventricular (AV) node block. The arrow shows a regular interval atrial, or P, wave, without a corresponding ventricular, or QRS, beat.

The patient was placed on continuous telemetry along with EEG monitoring in the epilepsy monitoring unit. On telemetry during awake and sleep periods, the patient had multiple sinus pauses of variable duration, ranging from 3 to 8.5 seconds. An EKG during these events showed a second-degree heart block (Figure 1).

The patient, however, remained conscious, unless they were already asleep, during these events. Subsequent workup with a transthoracic echocardiogram was unremarkable. A sleep study was also performed on the inpatient and showed mild obstructive sleep apnea, which was not thought to be causing the abnormal cardiac rhythm.

Given the severity of the heart block, pacemaker implantation was considered. Based on a review of case reports, VNS was identified as a potential source of the arrhythmia. The VNS was turned off, and his sinus pauses and second-degree heart block resolved completely. His lethargy gradually improved, and the patient was discharged home. A 14-day cardiac monitor after discharge revealed no persistent bradycardia or sinus pauses. With the complete and persistent resolution of the cardiac abnormalities after deactivating the VNS, the source of the arrhythmias was confirmed as due to VNS.

## Discussion

In the VNS clinical trial open-label follow-up study, discontinuation of VNS therapy was uncommon, and only about 5-16% of discontinuations were due to an adverse side effect [3]. Including all patients with VNS, serious adverse events due to VNS are rare, and cardiac arrhythmias are estimated to occur with VNS at 0.1% [7]. Transient cardiac arrhythmias have been reported most often in the perioperative period during lead testing: reasons for this are unclear but can result in serious arrhythmia, including heart block and asystole [7,8]. Arrhythmias can also occur late after VNS placement, up to nine years after VNS implantation [9,10]. Furthermore, there are now multiple case reports of life-threatening VNS device-induced cardiac arrhythmias, usually second-degree AV block with periods of asystole, occurring years after device implantation: all arrhythmias resolved after VNS deactivation except for one patient who required pacemaker implantation due to refusing any VNS adjustment [8-11]. It is possible that some events are underrecognized given their rarity and likely similar appearance to sudden unexplained death in epilepsy (SUDEP). SUDEP is thought to be related to seizure-induced cardiac arrhythmia, and the VNS population has an increased risk of SUDEP given medically refractory epilepsy. Thankfully, VNS device-induced cardiac arrhythmias are stopped with device discontinuation.

Cardiac arrhythmia-related syncopal events can be difficult to diagnose in patients with medically intractable epilepsy. Atonic seizures can look similar to cardiac syncope, given that both events involve sudden loss of body tone. VNS device-induced arrhythmias have been initially misdiagnosed as atonic seizures [12,13]. Cardiac arrhythmia testing is an important diagnostic consideration for any patient presenting with new-onset syncope or an atonic episode. Patients with epilepsy can have a risk for both epileptic atonic events and cardiogenic atonic events due to medications and neuromodulation devices like VNS, so the differential remains broad for new atonic events; furthermore, the use of telemetry during video EEG evaluation can evaluate both epileptic and cardiogenic causes simultaneously.

## Conclusions

Vagal nerve stimulation devices can reduce seizure severity and frequency, but they can be accompanied by rare, severe side effects. Cardiac arrhythmia, including heart block, is a possible side effect of VNS devices for medically refractory epilepsy, even when the device is placed on the left vagal nerve. Clinical events of sudden syncope can occur, which can mimic atonic seizures, making the clinical recognition of VNS-induced arrhythmias challenging. High clinical suspicion is required, along with prompt intervention, to prevent sudden cardiac arrest and death.
